# Supplementary material for: The Use of Levosimendan after Out-of-Hospital Cardiac Arrest and Its Association with Outcome—An Observational Study
Source: J Clin Med. 2022 May 6;11(9):2621. doi: 10.3390/jcm11092621 (PMC9103888; doi:10.3390/jcm11092621)
Supplement: Supplementary file 1 [file jcm-11-02621-s001.zip › Table S1a,b.pdf]

Supplementary Table S1a. Univariable and multivariable logistic regression analysis showing the association of different variables including levosimendan treatment (< 24 hours) and 30-day mortality in out-of-hospital-cardiac arrest patients admitted to the intensive care unit in Stockholm, Sweden, 2010-2016.

	Univariable		Multivariable	
Variable	**Odds Ratio (95% CI)**	**p-value**	**Odds Ratio (95% CI)**	**p-value**
Levosimendan < 24 hours	0.59 (0.38-0.90)	0.02	0.94 (0.56-1.57)	0.82
Male sex (=1)	0.54 (0.39-0.74)	< 0.001	0.84 (0.58-1.22)	0.37
Age, years	1.02 (1.01-1.03)	< 0.001	1.03 (1.02-1.04)	< 0.001
Shockable rhythm	0.12 (0.09-0.17)	< 0.001	0.13 (0.09-0.19)	< 0.001
Witnessed event	0.44 (0.31-0.63)	< 0.001	0.57 (0.38-0.87)	0.01
Bystander CPR	0.46 (0.35-0.62)	< 0.001	0.76 (0.54-1.07)	0.12
Myocardial infarction	0.41 (0.31-0.56)	< 0.001	0.87 (0.59-1.28)	0.48
Abbreviations: CI = confidence interval; CPR= cardiopulmonary resuscitation. Model area under the curve 0.80, Hosmer-Lemeshow p-value 0.40.				

Supplementary Table S1b. Univariable and multivariable logistic regression analysis showing the association of different variables including levosimendan treatment (< 24 hours) and 30-day mortality in out-of-hospital-cardiac arrest patients with initial shockable rhythm admitted to the intensive care unit in Stockholm, Sweden, 2010-2016.

	Univariable		Multivariable	
Variable	**Odds Ratio (95% CI)**	**p-value**	**Odds Ratio (95% CI)**	**p-value**
Levosimendan < 24 hours	1.45 (0.84-2.50)	0.18	1.35 (0.75-2.43)	0.32
Male sex (=1)	0.54 (0.39-0.74)	< 0.001	0.69 (0.40-1.20)	0.19
Age, years	1.02 (1.01-1.03)	< 0.001	1.03 (1.02-1.05)	< 0.001
Witnessed event	0.44 (0.31-0.63)	< 0.001	0.47 (0.25-0.86)	0.02
Bystander CPR	0.46 (0.35-0.62)	< 0.001	0.66 (0.42-1.06)	0.09
Myocardial infarction	0.41 (0.31-0.56)	< 0.001	1.11 (0.70-1.77)	0.65
Abbreviations: CI = confidence interval; CPR= cardiopulmonary resuscitation. Model area under the curve 0.67, Hosmer-Lemeshow p-value 0.20.				
