# Supplementary material for: The Use of Levosimendan after Out-of-Hospital Cardiac Arrest and Its Association with Outcome—An Observational Study
Source: J Clin Med. 2022 May 6;11(9):2621. doi: 10.3390/jcm11092621 (PMC9103888; doi:10.3390/jcm11092621)
Supplement: Supplementary file 1 [file jcm-11-02621-s001.zip › Table S2a,b.pdf]

Table S2a. Baseline characteristics and outcome before propensity score matching

	**Control**	**Levosimendan**	**p**	**SMD**
n	846	94		
Sex, male, n (%)	568 (67)	76 (81)	0.01	0.32
Age (median [IQR])	67 [56, 77]	66.00 [60, 74]	0.99	0.10
Witnessed arrest, n (%)	617 (73)	73 (78)	0.39	0.11
Shockable rhythm, n (%)	312 (37)	62 (66)	<0.001	0.61
Myocardial infarction, n (%)	199 (24)	44 (47)	<0.001	0.50
EMS response, minutes (median [IQR])	9 [7,13]	10 [6, 14]	0.76	0.07
Bystander CPR, n (%)	391 (46)	54 (57)	0.05	0.23
Dead at 30 days, n (%)	593 (70)	55 (59)	0.03	0.24

Table S2b. Baseline characteristics and outcome after propensity score matching

	Control	Levosimendan	p	test
n	93	93		
Sex, male, n (%)	83 (89)	75 (81)	0.15	
Age (median [IQR])	67 [58, 76]	66 [60, 74]	0.90	nonnorm
Witnessed arrest, n (%)	77 (83)	74 (80)	0.71	
Shockable rhythm, n (%)	51 (55)	61 (66)	0.18	
Myocardial infarction, n (%)	41 (44)	43 (46)	0.88	
EMS response, minutes (median [IQR])	9 [6, 13]	10 [7, 14]	0.94	nonnorm
Bystander CPR, n (%)	60 (65)	55 (59)	0.55	
Dead at 30 days, n (%)	56 (60)	54 (58)	0.88	
