# Supplementary material for: The Use of Levosimendan after Out-of-Hospital Cardiac Arrest and Its Association with Outcome—An Observational Study
Source: J Clin Med. 2022 May 6;11(9):2621. doi: 10.3390/jcm11092621 (PMC9103888; doi:10.3390/jcm11092621)
Supplement: Supplementary file 1 [file jcm-11-02621-s001.zip › Table S3a,b.pdf]

Supplementary Table S3a. Baseline characteristics in subgroups of time-to-treatment-start with levosimendan in out-of-hospital-cardiac arrest patients admitted to the intensive care unit in Stockholm, Sweden, 2010-2016.

Characteristics	Levosimendan < 6h (n=47)	p-value	Levosimendan 6-12h n=31	p-value	Levosimendan 12-24h (n=16)	p-value	No levosimendan (n=846)
Sex, n= (%)							
Male (=1)	37 (79)	0.10	24 (77)	0.23	15 (94)	0.02	568 (67)
Age, year							
Median	67 (62-74)	0.71	65 (55-75)	0.67	67 (59-75)	0.97	67 (56-77)
Acute myocardial ischemia, n= (%)							
Myocardial infarction, any	22 (47)	<0.001	14 (45)	0.01	8 (50)	0.01	199 (24)
STEMI	19 (86)	0.002	10 (71)	0.15	4 (50)	0.92	103 (52)
CPR characteristics n= (%)							
Shockable rhythm	30 (64)	<0.001	20 (65)	0.002	12 (75)	0.01	311 (37)
Witnessed event*	34/44 (77)	0.46	23 (74)	0.81	14/15 (93)	0.07	574/795 (72)
Bystander CPR *	31/44 (71)	0.002	10 (32)	0.13	12/15 (80)	0.01	364/792 (46)
Respons-time, minutes median (IQR)*	9 (6-14)	0.84	11/25 (8-18)	0.16	8 (5-12)	0.19	9 (7-13)
Coronary angiography + PCI	18 (38)	<0.001	11(36)	<0.001	8 (50)	<0.001	112 (13)

Data are presented as median (IQR) for continuous measures, and n (%) for categorical measures. Abbreviations: OHCA= out-of-hospital-cardiac arrest; ICU = intensive care unit; STEMI= ST-elevation myocardial infarction; CPR= cardiopulmonary resuscitation; PCI= percutaneous coronary intervention. * missing values. In the variables with missing valus we present nominator-denominator.

Supplementary Table S3b. Interventions and outcomes in subgroups of time-to-treatment-start with levosimendan in out-of-hospital-cardiac arrest patients admitted to the intensive care unit in Stockholm, Sweden, 2010-2016

**Characteristics**	Levosimendan < 6hours n=47	p-value	Levosimendan 6-12h n=31	p-value	Levosimendan 12-24h n=16	p-value	No levosimendan n=846
ICU-interventions and outcome							
Vasoactive / inptropic support, n= (%)							
Noradrenaline	46 (98)	<0.001	30 (97)	<0.001	16 (100)	<0.001	515 (61)
Adrenaline	9 (19)	0.01	4 (13)	0.16	1 (6)	0.97	55 (7)
Amiodarone	6 (13)	0.02	6 (19)	<0.001	3 (19)	0.01	40 (5)
Milrinone	9 (19)	<0.001	1 (3)	0.12	4 (25)	<0.001	6 (1)
Dobutamine	7 (15)	0.08	6 (19)	0.02	5 (31)	<0.001	65 (8)
Arginine vasopressin	0 (0)	0.50	1 (3)	0.22	0 (0)	0.70	8 (1)
Cardiogenic shock *	7/30 (23)	0.30	3/18 (17)	0.94	3/10 (30)	0.24	52/326 (16)
TTM *	21/35 (60)	0.01	16/24 (67)	0.01	8/12 (67)	0.04	237/629 (38)
ICU stay days, median (IQR)*	3 (2-6)	0.01	3 (2-6)	0.01	6 (4-9)**	<0.001	2 (1-4)
Mortality, 30-days, n= (%)	21 (45)	<0.001	24 (77)	0.36	9 (56)	0.25	590 (70)

Data are presented as median (IQR) for continuous measures, and n (%) for categorical measures. Abbreviations: ICU = intensive care unit; TTM= targeted temperature management. * missing values. In the variables with missing valus we present nominator-denominator.
